# Metabolically Healthy Obesity and the Risk of Cardiovascular Disease in the Elderly Population

**DOI:** 10.1371/journal.pone.0154273

**Published:** 2016-04-21

**Authors:** Klodian Dhana, Chantal M. Koolhaas, Elisabeth F. C. van Rossum, M. Arfan Ikram, Albert Hofman, Maryam Kavousi, Oscar H. Franco

**Affiliations:** 1 Department of Epidemiology, Erasmus Medical Center, Rotterdam, The Netherlands; 2 Department of Internal Medicine, Erasmus Medical Center, Rotterdam, The Netherlands; 3 Department of Neurology Erasmus Medical Center, Rotterdam, The Netherlands; 4 Department of Radiology Erasmus Medical Center, Rotterdam, The Netherlands; 5 Department of Epidemiology, Harvard T.H. Chan School of Public Health, Boston, MA, United States of America; Shanghai Institute of Hypertension, CHINA

## Abstract

**Background:**

Whether being metabolically healthy obese (MHO)—defined by the presence of obesity in the absence of metabolic syndrome—is associated with subsequent cardiovascular disease (CVD) remains unclear and may depend on the participants’ age. We examined the association of being MHO with CVD risk in the elderly.

**Methods and Findings:**

This study included 5,314 individuals (mean age 68 years) from the prospective population-based Rotterdam Study. We categorized our population in groups according to body mass index (BMI) and presence and absence of metabolic syndrome, and estimated the hazard ratio (HR) and 95% confidence interval (95%CI) for every group by using Cox proportional hazard models. Among 1048 (19.7%) obese individuals we identified 260 (24.8%) MHO subjects. Over 14 years of follow-up there were 861 incident CVD cases. In the multivariable adjusted analysis, we did not observe an increased CVD risk in MHO individuals (HR 1.07, 95%CI 0.75–1.53), compared to normal weight individuals without metabolic syndrome. CVD risk was increased by the presence of metabolic syndrome in normal weight (HR 1.35, 95%CI 1.02–1.80), overweight (HR 1.32, 95%CI 1.09–1.60) and obese (HR 1.33, 95%CI 1.07–1.66) individuals, compared to those with normal weight without metabolic syndrome. In a mediation analysis, 71.3% of the association between BMI and CVD was explained by the presence of metabolic syndrome.

**Conclusions:**

In our elderly population, we found that the presence of obesity without metabolic syndrome did not confer a higher CVD risk. However, metabolic syndrome was strongly associated with CVD risk, and was associated with an increased risk in all BMI categories. Therefore, preventive interventions targeting cardiometabolic risk factors could be considered in elderly, regardless of weight status.

## Introduction

Although obesity in young individuals is an established risk factor for cardiovascular disease (CVD), the effect of obesity in the elderly seems to dilute with advancing age, rising towards controversial discussions [1, 2]. The discrepancy between the findings at younger versus older ages suggests that additional factors may alter the effect of obesity on risk of CVD. It is well known that the presence of metabolic syndrome (a cluster of cardiovascular risk factors including hypertension, dyslipidemia, hyperglycemia and abdominal obesity) differs among individuals with similar body mass index (BMI), which indicates that the risk of CVD within specific categories of BMI could be heterogeneous [3, 4]. In this context, recent interest has focused on a subgroup of obese individuals, termed the metabolically healthy obese (MHO), who despite their increased BMI (BMI≥30kg/m^2^) seem to have an adequate metabolic profile and do not have metabolic syndrome[3, 5].

The effect of being MHO on health outcomes remains controversial. While some studies have reported no increased risk of CVD among MHO individuals [4, 5–7], several other studies have shown an increased risk of CVD in this group [8–11]. For example, a 17 year follow-up study of adults aged 39–63 years found an increased CVD risk for MHO individuals [8]. In contrast, another study in women of 45 years and older, with 10 years follow-up, found no increased CVD risk for obese individuals without metabolic syndrome [7]. It is important to note that in these studies the mean age was below 65 years, indicating that information among the elderly is scarce. In the elderly, the relation between body weight, body composition, and health behaviors is different than in younger adults [12, 13]. Therefore, the impact of being MHO could differ between younger, middle-aged and elderly adults.

In the current study, we aimed to study the role of being MHO in association with risk of CVD in middle-aged and elderly individuals. We sought to examine the association of metabolic syndrome with CVD among different BMI categories and to examine the contribution of metabolic syndrome to the association between BMI and CVD.

## Methods

### Study design, setting, and population

This study was embedded within the Rotterdam Study (RS), a prospective population-based cohort study among subjects aged 55 years or older in the municipality of Rotterdam, the Netherlands [14]. The baseline examination of the initial cohort (RS-I) was completed between 1990 and 1993. In 2000–2001, the Rotterdam Study was extended (RS-II) with 3,011 participants who had become ≥ 55 years old or had moved into the study district. For the current study, we used data from the participants attending the third examination of the original cohort (RS-I visit 3, between 1997 and 1999; n = 4,797) and the participants attending the first examination of the second cohort (RS-II visit 1, between 2000 and 2001; n = 3,011). We excluded all participants with a history of CVD (coronary heart disease, cerebrovascular disease, or heart failure) at baseline (n = 1,505), those who did not visit the research center at baseline for assessment of cardiovascular risk factors or BMI (n = 660), those who did not have fasting plasma measurements (n = 289), and finally those who were underweight (BMI <18.5 kg/m^2^) at baseline (n = 40). This left a total of 5,314 individuals eligible for the present analyses. The Rotterdam Study has been approved by the Medical Ethics Committee of the Erasmus MC and by the Ministry of Health, Welfare and Sport of the Netherlands, implementing the “Wet Bevolkingsonderzoek: ERGO (Population Studies Act: Rotterdam Study)”. All participants provided written informed consent to participate in the study and to obtain information from their treating physicians. Detailed information on the design of the Rotterdam Study can be found elsewhere [14].

### Assessment of anthropometric, lifestyle exposures, and laboratory measurements

Height and weight were measured with the participants standing without shoes and heavy outer garments. BMI was calculated as weight divided by height squared (kg/m^2^). Waist circumference was measured at the level midway between the lower rib margin and the iliac crest with participants in standing position without heavy outer garments and with emptied pockets, breathing out gently. Information on education (≥ high school, < high school), smoking status (current or former/never), alcohol use (drinking alcohol or not), and physical activity[15] were obtained through interview. Blood pressure was measured in seated position and averaged across two measures. Fasting triglycerides, total cholesterol and high-density lipoprotein (HDL) cholesterol and glucose levels were measured using standard laboratory techniques [16, 17]. To assess kidney function we estimated the glomerular filtration rate (GFR), using the Chronic Kidney Disease Epidemiology Collaboration CKD-EPI equation [18].

### Metabolic syndrome and body mass index

Normal weight (18.5–25 kg/m^2^), overweight (25–29.9 kg/m^2^), and obese participants (≥ 30 kg/m^2^) were categorized as either with or without metabolic syndrome based on the “Harmonized metabolic syndrome definition” [19]. Participants were considered to have metabolic syndrome if they had ≥ 3 of the following five components: (1) waist circumference ≥ 102 cm in men and ≥ 88 cm in women; (2) systolic blood pressure ≥ 130 mmHg and/or diastolic blood pressure ≥ 85 mmHg or use of antihypertensive treatment; (3) fasting plasma triglycerides ≥ 150 mg/dL; (4) HDL cholesterol level < 40 mg/dL in men and < 50 mg/dL in women; (5) elevated fasting glucose ≥ 100 mg/dL or treatment for diabetes mellitus. Consequently, this information was used to create six phenotypes: normal weight, overweight and obese with or without metabolic syndrome. MHO was defined as obesity without metabolic syndrome (i.e. BMI ≥ 30kg/m^2^ and having ≤ 2 components of metabolic syndrome).

### Definition of outcome

The main outcome measure under study was incident hard atherosclerotic CVD, composed of fatal and non-fatal myocardial infarction, other coronary heart disease mortality, and fatal and non-fatal stroke [20]. Definite and possible fatal coronary heart disease events are coded by using the definitions applied within the Cardiovascular Health Study and Atherosclerosis Risk in the Communities Study [21]. Stroke is defined as a syndrome of rapidly developing clinical signs of focal (or global) disturbance of cerebral function, with symptoms lasting 24 hours or longer or leading to death, with no apparent origin other than vascular [22]. Data on incident CVD is collected using an automated follow-up system, through gathering information from general practitioners in the study area and subsequent collection of information from letters of medical specialists and discharge reports in case of hospitalization. A consensus panel, led by a physician with expertise in field, adjudicated the diagnosis using standardized definitions. The follow-up was complete until January 1, 2012.

### Statistical analysis

Baseline characteristics of the study population are presented as mean ± SD (or frequency and percentage when appropriate) for the 6 phenotypes formed by the metabolic syndrome across different BMI categories. In our main analysis, we used Cox proportional hazard regression analysis to estimate the hazard ratio (HR) and 95% confidence interval (95%CI) for the six phenotypes described above in association with CVD, using normal weight without metabolic syndrome as the reference category. Additionally, we separately estimated the HR and 95%CI for the associations of the BMI categories and metabolic syndrome with CVD. Proportional hazards assumptions were confirmed in all Cox models, by visually comparing the Kaplan-Meier curves of the different groups. The models were adjusted for age, gender, smoking, cholesterol level, lipid-lowering medication use, GFR, alcohol use, education and physical activity. We decided a priori not to adjust for systolic blood pressure, triglycerides, HDL cholesterol, glucose, diabetes mellitus, and waist circumference, as they are all part of the definition of metabolic syndrome [9]. Kaplan Meier analyses and log rank tests were used to build plots for CVD incidence trends among the BMI categories, metabolic syndrome status and the joint BMI and metabolic syndrome phenotypes. We did not observe a significant association of gender with either BMI, metabolic syndrome or the joint BMI and metabolic syndrome phenotypes. Moreover, we did not find an interaction between BMI and metabolic syndrome.

In a mediation analysis, we examined whether metabolic syndrome could be considered a mediator in the association between BMI and CVD risk. The percentage of excess risk mediated was calculated as [(HR_con adj_ − HR_con + med adj_)/(HR_con adj_ − 1)] × 100%, where HR_con adj_ is the confounder-adjusted HR for CVD and HR_con + med adj_ is the confounder and mediator—adjusted HR [23].

#### Sensitivity analyses

Due to the high competing risk of non-CVD death among the elderly, we performed a competing risk analysis using the method proposed by Fine and Gray [24]. Additionally, we repeated the main analysis in participants older than 65, to specifically examine the associations in the elderly. Although there was no interaction between BMI or metabolic syndrome with gender, we repeated our main analysis in men and women, because of gender-differences in body fat distribution. Moreover, to show the independence of metabolic syndrome over BMI, we adjusted for BMI (continuously and categorical) in the association of metabolic status with CVD. Finally, to investigate the dose-response relation between metabolic syndrome and CVD, we evaluated the risk of CVD according to the presence of one, two, three, four, or five components of the metabolic syndrome.

Co-variables were missing in less than 5% of the participants, with the exception of treatment for hypertension, which had 7.7% missing. We used the single imputation by the Expectation Maximization method in SPSS. The analyses were performed using IBM SPSS Statistics for Windows (IBM SPSS Statistics for Windows, Armonk, NY: IBM Corp) and R version 3.1.3 (R Foundation for Statistical Computing, Vienna, Austria).

## Results

Characteristics of the study participants, stratified by BMI and metabolic syndrome, are shown in Table 1. Among all participants, 57.2% (n = 3,038) were without metabolic syndrome and 19.7% (n = 1,048) were obese. The MHO phenotype represented 4.9% (n = 260) of the total study population and 24.8% of the obese population. MHO subjects were more often women and reported more physical activity compared to obese individuals with metabolic syndrome. During a median follow-up of 10.3 years (interquartile range: 8.1–11.7 years), there were 861 (16.2%) incident CVD events.

**Table 1:** Baseline characteristics of study population across the categories of metabolic health status and body mass index.

	No metabolic syndrome (n = 3038)			Metabolic syndrome (n = 2276)		
	Normal weight	Overweight	Obese	Normal weight	Overweight	Obese
**n, %**	1444 (47.5)	1334 (43.9)	260 (8.6)	306 (13.4)	1182 (51.9)	788 (34.7)
**Women, %**	859 (59.5)	686 (51.4)	203 (78.1)	197 (64.4)	689 (58.3)	568 (72.1)
**Age, years**	68.2 ± 8.0	68.1 ± 8.0	68.3 ± 8.1	70.1 ± 8.5	68.8 ± 8.1	68.1 ± 7.9
**High education, %**	520 (36.1)	552 (41.3)	132 (50.7)	124 (40.5)	498 (42.1)	381 (48.3)
**Current smokers, %**	346 (24.0)	226 (16.9)	40 (15.4)	92 (30.1)	254 (21.5)	117 (14.8)
**Alcohol use, %**	868 (60.1)	721 (54.0)	177 (68.1)	194 (63.4)	690 (58.4)	554 (70.3)
**Physical activity, METHour/week**	86.6 ± 42.9	87.1 ± 45.5	85.3 ± 44.1	81.7 ± 43.4	82.0 ± 40.7	80.4 ± 44.0
**Estimated glomerular filtration rate, GFR**	77.2 ± 13.6	76.3 ± 13.5	76.4 ± 15.7	73.1 ± 15.4	74.1 ± 14.7	75.4 ± 15.2
**Waist circumference, cm**	83.2 ± 8.3	92.1 ± 8.0	100.3 ± 9.4	89.1 ± 8.2	97.3 ± 7.5	107 ± 10.0
**BMI, kg/m**^**2**^	23.0 ± 1.5	26.9 ± 1.3	32.3 ± 2.3	23.6 ± 1.2	27.6 ± 1.4	33.4 ± 3.1
**Triglycerides, mg/dl**	103.1 ± 39.2	109.1 ± 42.3	107.6 ± 30.7	181.1 ± 79.3	173.2 ± 77.0	172.4 ± 86.2
**Fasting glucose, mg/dl**	97.5 ± 14.7	100.0 ± 16.7	98.6 ± 15.1	115.8 ± 34.5	114.5 ± 28.6	120.3 ± 33.5
**HDL cholesterol, mg/dl**	61.2 ± 14.7	57.5 ± 13.4	59.7 ± 13.8	46.1 ± 15.1	46.7 ± 12.1	48.2 ± 14.0
**Systolic blood pressure, mmHg**	137.1 ± 21.3	139.9 ± 20.6	140.3 ± 23.1	150 ± 19.7	149.4 ± 19.8	149.4 ±18.8
**Diastolic blood pressure, mmHg**	74.2 ± 10.7	76.4 ± 10.6	77.2 ± 10.5	77.1 ± 10.5	79.2 ± 10.9	79.9 ± 10.9
**Treatment for hypertension, n (%)**	154 (10.7)	231 (17.3)	73 (28.1)	64 (20.9)	376 (31.8)	325 (41.2)
**Total cholesterol, mg/dl**	224.4 ± 36.0	227.4 ± 36.4	228.3 ± 35.9	232.8 ± 39.2	229.7 ± 37.8	226.6 ± 37.0
**Treatment for hyperlipidemia, n (%)**	82 (5.7)	93 (7.0)	16 (6.2)	50 (16.3)	158 (13.4)	111 (14.1)

n, number; MET, metabolic equivalent of task; BMI, body mass index; HDL, high-density lipoprotein.

Values are means ± standard deviation or numbers (percentages).

In the association between BMI categories and CVD, we found that being overweight (HR 1.12, 95%CI: 0.96–1.30) or obese (HR 1.18, 95% CI: 0.97–1.44) was not significantly associated with risk of CVD, compared to being normal weight. In contrast, individuals with metabolic syndrome had an increased risk of CVD (HR: 1.27, 95% CI: 1.11–1.46), compared to individuals without metabolic syndrome.

**Fig 1:**
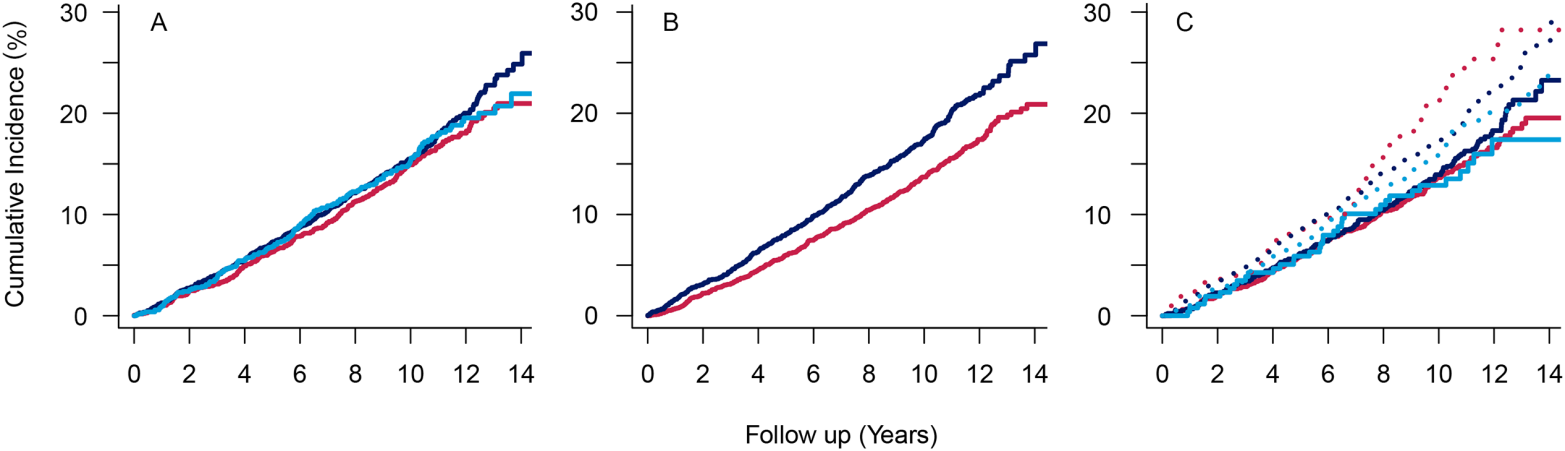
Cumulative incidence of cardiovascular disease as a function of follow-up time according to body mass index categories (A), metabolic syndrome (B) and the joint body mass index and metabolic syndrome phenotypes (C). (A) Red: “normal weight” (1,750 individuals), dark blue: “overweight” (2,516 individuals), light blue: “obese” (1,048 individuals).These incident curves do not differ significantly from each other over the follow-up (log-rank test, P = 0.395). (B) Red: “without metabolic syndrome” (3,038 individuals), dark blue: “with metabolic syndrome” (2276 individuals).These incident curves differ significantly from each other over the follow-up (log-rank test, P = 0.001). (C) Solid lines indicate individuals without metabolic syndrome and dotted lines individuals with metabolic syndrome. Red: “normal weight” (including 1444 without metabolic syndrome and 306 with metabolic syndrome); dark blue: “overweight” (including 1334 without metabolic syndrome and 1182 with metabolic syndrome); light blue: “obese” (including 260 without metabolic syndrome and 788 with metabolic syndrome). These incident curves differ significantly from each other over the follow-up (log-rank test, P = 0.001).

In Table 2, we present the HRs (95% CIs) for the association between the joint BMI and metabolic syndrome phenotypes with incident CVD. Compared to normal weight subjects without metabolic syndrome, the HRs (95%CIs) were 1.08 (0.89–1.32) in overweight and 1.07 (0.75–1.53) in obese subjects without metabolic syndrome and 1.35 (95%CI, 1.02–1.80) in normal weight, 1.32 (1.09–1.60) in overweight and 1.33 (1.07–1.66) in obese subjects with metabolic syndrome. Fig 1 presents the Kaplan-Meier survival curves of the cumulative incidence of CVD by categories of BMI (Fig 1A), by presence of metabolic syndrome (Fig 1B) and as a function of the joint BMI and metabolic status phenotypes (Fig 1C). The cumulative incidences of CVD were not different among categories of BMI (log-rank trend P = 0.395). However, the cumulative incidence was higher in individuals with metabolic syndrome compared to those without (log-rank trend P<0.001). As expected, the cumulative incidence of CVD was higher in normal weight, overweight and obese individuals with metabolic syndrome, than in individuals without metabolic syndrome (log-rank trend P < 0.001). In the mediation analysis (Table 3), the percentage of excess risk mediated by metabolic syndrome in the association between BMI (as a continuous variable) and CVD was 71.3%; that is, 71.3% of the association between BMI and CVD could be explained by metabolic syndrome. By categorizing BMI, this proportion increased up to 73.1%.

**Table 2:** Association of the joint body mass index and metabolic syndrome phenotypes with cardiovascular disease.

	N	Events	HR (95%CI)
**No metabolic syndrome**			
normal weight	1444	203	1 (Reference)
overweight	1334	205	1.08 (0.89–1.32)
obese	260	36	1.07 (0.75–1.53)
**Metabolic syndrome**			
normal weight	306	63	1.35 (1.02–1.80)
overweight	1182	219	1.32 (1.09–1.60)
obese	788	135	1.33 (1.07–1.66)

N, number; HR, hazard ratio; CI, confidence interval.

Hazard ratios and 95%CI are for the multivariable model adjusted for age, gender, smoking, total cholesterol, treatment for hyperlipidemia, estimated glomerular filtration rate (GFR), alcohol, physical activity and education.

**Table 3:** Percentage of excess risk mediated by metabolic syndrome in association between body mass index and cardiovascular disease.

Exposure	Mediator	HR confounder adjusted	HR confounder and mediator adjusted	PERM, %
BMI (continuously)	Metabolic syndrome	1.0174	1.0053	71.3
BMI (categorically)	Metabolic syndrome	1.0901	1.0242	73.1

HR, hazard ratio; PERM, percentage of excess risk mediated; BMI, body mass index

Percentage of excess risk mediated (PERM) was calucalted as ((HR_confounder adjusted_−HR_confounder+mediator adjusted_)/(HR_confounder adjusted_ -1))*100%. Hazard ratios are for the multivariable model adjusted for age, gender, smoking, cholesterol, treatment for hyperlipidemia, estimated glomerular filtration rate (GFR), alcohol, physical activity and education. Body mass index was categorized as normal weight (18.5–24.9 kg/m^2^), overweight (25–29.9 kg/m^2^), and obese (≥30kg/m^2^).

### Sensitivity analyses

S1 Table shows that the HRs (95%CIs) from the competing risk approach were not substantially different from our original analysis. Additionally, when we repeated the main analysis in adults 65 and older, or in men and women separately, we found similar results (S2 and S3 Tables). Moreover, the association of metabolic status with CVD did not largely change after we further adjusted for BMI (S4 Table). The new HR (95%CI) of the presence of metabolic syndrome was 1.25 (1.08–1.46), when we adjusted for BMI continuously. Finally, when we examined the dose-response relation between metabolic syndrome components and CVD, we observed that the risk of CVD increased stepwise according to the presence of one, two, three, four, or five components of metabolic syndrome (S5 Table).

## Discussion

In this population-based study of 5,314 middle-aged and elderly individuals, 19.7% of the population was obese, of which 24.8% were without metabolic syndrome (i.e. MHO). Our study yields three key findings. First, compared to normal weight individuals without metabolic syndrome, MHO subjects were not at significant increased risk of CVD. Second, regardless of being normal weight, overweight or obese, the presence of metabolic syndrome consistently increased CVD risk. Third, 71.3% of the association between BMI and CVD was explained by metabolic syndrome. These findings highlight the importance of assessing CVD risk irrespective of BMI in an older population, and stress the importance of metabolic syndrome in the elderly.

Previous studies evaluating the association between metabolically healthy obesity and CVD risk have shown inconsistent results [4, 5–11]. While some studies reported no increased risk of CVD among MHO individuals [4, 5–7], several other studies have shown an increased CVD risk in this group [8–11]. In agreement with our study, Meigs et al., in an 11-year follow-up study of 2,902 men and women (mean age of 53 years), reported that MHO individuals do not have an increased risk of CVD in the Framingham Offspring Study [5]. Similarly, a report from the Women’s Ischemia Syndrome Evaluation (WISE) study showed that the presence of metabolic syndrome, but not BMI, predicted 3-year risk of cardiovascular death, in 21 to 86-year-old women referred for angiography [4]. Moreover, a large prospective study of 25,626 women aged 45 years and older, followed up to 10 years, found that MHO individuals were not at increased risk of CVD [7]. Additionally, this study showed that the presence of metabolic syndrome conferred a higher risk of developing CVD than BMI [7]. In contrast with these studies, Hinnouho et al., in a 17 year follow-up study, including men and women aged 35–55 years, found that MHO individuals were at increased risk of incident CVD, compared with normal weight individuals without metabolic syndrome [8]. Moreover, in contrast to our findings, these authors revealed a gradual increased CVD risk for overweight and obese individuals, compared to normal weight persons, both in individuals with and without metabolic syndrome. Similarly to Hinnouho et al, Thomsen et al reported that MHO individuals are at higher risk of developing myocardial infarction and ischemic heart disease [9]. Additionally, this short follow-up (median 3.6 years) study of 71,527 men and women aged 20–100 years showed that both in individuals with and without metabolic syndrome, there were increasing cumulative incidences of myocardial infarction and ischemic heart disease going from normal weight through overweight to obesity. Furthermore, in their study, metabolic syndrome explained only 12% of the risk attributed to BMI in the association with myocardial infarction and ischemic heart disease, whereas in our study metabolic syndrome explained 71.3% of the risk attributed to BMI in association with CVD.

The different findings regarding the association between metabolically healthy obesity and CVD risk in the studies mentioned above could reflect differences in the age range of the participants included in the different studies. To explain the importance of age in the association between BMI, metabolic syndrome and CVD, two possibilities could be considered. First, studies have shown that the magnitude of the relation between BMI and CVD risk weakens with age [25–27]. Indeed, in our study there was no evidence for a dose-response increase in CVD risk within BMI categories in individuals with or without metabolic syndrome, whereas other studies conducted in younger populations showed a progressive increase in CVD risk, going from normal weight through overweight to obesity [8, 9]. Although body weight and BMI may remain relatively unchanged with advancing age, there is a change in body composition, followed by visceral fat increases and muscle mass decreases [28]. Hereby, elderly individuals can be considered overweight by body fat standards, without having a BMI above 25. Consequently, BMI becomes a less accurate reflection of fat mass [29] and BMI alone may therefore not be a precise predictor of cardiovascular risk in elderly. On the other hand, metabolic syndrome is an established predictor of future CVD, and as we also showed in our study, the increased risk of CVD starts with the presence of just one component of the metabolic syndrome. Moreover, the prevalence of metabolic syndrome increases in older individuals [30] and was 42.8% in our population. Consequently, the metabolic syndrome becomes a more relevant condition in the elderly. Taken together, these findings stress the importance of metabolic syndrome over BMI in the development of future CVD among older adults, compared to young adults.

The mechanisms underlying the healthy metabolic profile of metabolically healthy obesity are still unclear. It has been suggested that the location, metabolic activity and histological characteristics of adipose tissue may determine metabolic health among obese individuals, whereas the amount of adipose tissue is less crucial [31]. In addition, the amount of years that an individual has been obese might play a role [32]. Moreover, studies have shown that MHO individuals have lower levels of C-reactive protein [33] and higher levels of insulin sensitivity, compared to obese individuals with metabolic syndrome [34]. Additionally, there is some evidence that obese individuals with metabolic syndrome are less fit than MHO individuals [35]. In our study, MHO participants had higher levels of physical activity than their obese counterparts with metabolic syndrome, which supports this last statement. However, a previous study has proposed that MHO is not a permanent state of the healthy metabolic profile, but rather a transient phase, moving toward glucose-metabolic abnormalities [8]. Therefore, it might be wise to re-evaluate the metabolic status of MHO individuals on a regular basis.

In our study, the presence of metabolic syndrome in normal weight individuals conferred a higher risk of CVD compared to obese individuals without metabolic syndrome, when we compared both groups to normal weight individuals without metabolic syndrome. Moreover, normal weight individuals with metabolic syndrome had a higher cumulative incidence of CVD than obese individuals with metabolic syndrome. Our study showed that the presence of metabolic syndrome in normal weight individuals was accompanied by a higher smoking prevalence and lower levels of physical activity, compared to obese individuals without metabolic syndrome. This finding is in accordance with a previous study [3], that showed that MHO individuals were more often nonsmokers and met the current physical activity guidelines more frequently than normal weight individuals with metabolic syndrome. Our observation that the cumulative incidence of CVD was higher in normal weight subjects with metabolic syndrome, compared to their obese counterparts, could be explained by the fact that normal weight individuals with metabolic syndrome were older and more often smokers than obese individuals with metabolic syndrome. Moreover, we found that normal weight individuals with metabolic syndrome had a lower proportion of treatment for hypertension at the baseline compared to obese individuals with metabolic syndrome. Remarkably, the baseline systolic blood pressure in both groups was similar. This may suggest that the obese individuals are more likely to be screened for CVD and subsequently receive medication.

Strengths of the current study include the prospective study design, large sample size, long follow-up, high follow-up rate, reliable assessment of CVD events and detailed assessment of lifestyle factors, components of the metabolic syndrome and cardiovascular risk factors. However, several limitations should be considered. First, our conclusions are drawn from the baseline measurements. In our analyses, we were not able to account for changes in BMI and metabolic factors during follow-up. Therefore, a degree of misclassification, due to changes in these risk factors over time might have occurred. Second, our study population included only Caucasian men and women above 55 years. Therefore, results from the present study cannot be generalized to other age-groups or ethnics groups.

To conclude, in our population-based study of middle-aged and elderly adults, MHO individuals were not at increased risk of CVD. However, the presence of the metabolic syndrome was associated with future cardiovascular risk similarly in normal weight, overweight and obese individuals Additionally, we showed that the association between BMI and CVD was largely (73.1%) explained by the presence of metabolic syndrome. Although it remains prudent to recommend weight loss in overweight and obese individuals and the benefits that these interventions can achieve expand beyond cardiovascular events, our results suggest that preventive interventions targeting cardiometabolic risk factors in older individuals should be considered regardless of weight status.

## Supporting Information

S1 Table Association of the joint body mass index and metabolic syndrome phenotypes with cardiovascular disease, adjusted for competing risk of mortality. Hazard ratios and 95% confidence intervals are presented for the multivariable model, adjusted for age, gender, smoking, cholesterol, treatment for hyperlipidemia, estimated glomerular filtration rate (GFR), alcohol, physical activity and education.
(DOCX)

S2 Table Association of the joint body mass index and metabolic syndrome phenotypes with cardiovascular disease in adults older than 65 years (n = 3,174). Hazard ratios and 95%confidence intervals are presented for the multivariable model, adjusted for age, gender, smoking, cholesterol, treatment for hyperlipidemia, estimated glomerular filtration rate (GFR), alcohol, physical activity and education.
(DOCX)

S3 Table Association of the joint body mass index and metabolic syndrome phenotypes with cardiovascular disease in men and women. Hazard ratios and 95%confidence intervals are presented for the multivariable model, adjusted for age, smoking, cholesterol, treatment for hyperlipidemia, estimated glomerular filtration rate (GFR), alcohol, physical activity and education.
(DOCX)

S4 Table Association of metabolic syndrome with cardiovascular disease after adjusting for body mass index. Hazard ratios and 95%confidence intervals are presented for the multivariable model adjusted for age, gender, smoking, cholesterol, treatment for hyperlipidemia, estimated glomerular filtration rate (GFR), alcohol, physical activity, education and body mass index.
(DOCX)

S5 Table Associations of one, two, three, four, or five components of the metabolic syndrome with cardiovascular disease. Hazard ratios and 95%confidence intervals are presented for the multivariable model adjusted for age, gender, smoking, cholesterol, treatment for hyperlipidemia, estimated glomerular filtration rate (GFR), alcohol, physical activity, and education.
(DOCX)
