# Supplementary material for: Metabolically Healthy Obesity and the Risk of Cardiovascular Disease in the Elderly Population
Source: PLoS One. 2016 Apr 21;11(4):e0154273. doi: 10.1371/journal.pone.0154273 (PMC4839559; doi:10.1371/journal.pone.0154273)
Supplement: S1 Table — Hazard ratios and 95% confidence intervals are presented for the multivariable model, adjusted for age, gender, smoking, cholesterol, treatment for hyperlipidemia, estimated glomerular filtration rate (GFR), alcohol, physical activity and education. (DOCX) [file pone.0154273.s001.docx]

**S1 Table. Association of the joint body mass index and metabolic syndrome phenotypes with cardiovascular disease, adjusted for competing risk of mortality.**

|  | | **N** | **Event** | **HR (95%CI)** |
| --- | --- | --- | --- | --- |
| **No metabolic syndrome** | normal weight | 1481 | 208 | 1 [Reference] |
|  | overweight | 1334 | 205 | 1.12 (0.92-1.37) |
|  | obese | 260 | 36 | 1.08 (0.76-1.55) |
| **Metabolic syndrome** | normal weight | 309 | 64 | 1.28 (0.96-1.72) |
|  | overweight | 1182 | 219 | 1.31 (1.08-1.60) |
|  | obese | 788 | 135 | 1.37 (1.09-1.71) |

Hazard ratios and 95%CI are for the multivariable model adjusted for age, gender, smoking, cholesterol, treatment for hyperlipidemia, estimated glomerular filtration rate (GFR), alcohol, physical activity and education.
