# Supplementary material for: Metabolically Healthy Obesity and the Risk of Cardiovascular Disease in the Elderly Population
Source: PLoS One. 2016 Apr 21;11(4):e0154273. doi: 10.1371/journal.pone.0154273 (PMC4839559; doi:10.1371/journal.pone.0154273)
Supplement: S2 Table — Hazard ratios and 95%confidence intervals are presented for the multivariable model, adjusted for age, gender, smoking, cholesterol, treatment for hyperlipidemia, estimated glomerular filtration rate (GFR), alcohol, physical activity and education. (DOCX) [file pone.0154273.s002.docx]

**S2 Table. Association of the joint body mass index and metabolic syndrome phenotypes with cardiovascular disease in adults older than 65 years (n=3174)**

|  | | **N** | **Event** | **HR (95%CI)** |
| --- | --- | --- | --- | --- |
| **No metabolic syndrome** | normal weight | 846 | 157 | 1 [Reference] |
|  | overweight | 770 | 168 | 1.14 (0.92-1.42) |
|  | obese | 158 | 32 | 1.15 (0.78-1.68) |
| **Metabolic syndrome** | normal weight | 203 | 51 | 1.32 (0.96-1.82) |
|  | overweight | 732 | 171 | 1.30 (1.05-1.63) |
|  | obese | 465 | 108 | 1.34 (1.04-1.72) |

Hazard ratios and 95%CI are for the multivariable model adjusted for age, gender, smoking, cholesterol, treatment for hyperlipidemia, estimated glomerular filtration rate (GFR), alcohol, physical activity and education.
