# Supplementary material for: Metabolically Healthy Obesity and the Risk of Cardiovascular Disease in the Elderly Population
Source: PLoS One. 2016 Apr 21;11(4):e0154273. doi: 10.1371/journal.pone.0154273 (PMC4839559; doi:10.1371/journal.pone.0154273)
Supplement: S3 Table — Hazard ratios and 95%confidence intervals are presented for the multivariable model, adjusted for age, smoking, cholesterol, treatment for hyperlipidemia, estimated glomerular filtration rate (GFR), alcohol, physical activity and education. (DOCX) [file pone.0154273.s003.docx]

**S3 Table. Association of the joint body mass index and metabolic syndrome phenotypes with cardiovascular disease in men and women**

| **Men (n=2112)** | | **N** | **Events** | **HR (95%CI)** |
| --- | --- | --- | --- | --- |
| **No metabolic syndrome** | normal weight | 585 | 98 | 1 (Reference) |
|  | overweight | 648 | 111 | 1.10 (0.83-1.44) |
|  | obese | 57 | 7 | 1.09 (0.50-2.35) |
| **Metabolic syndrome** | normal weight | 109 | 22 | 1.22 (0.76-1.94) |
|  | overweight | 493 | 106 | 1.42 (1.07-1.87) |
|  | obese | 220 | 40 | 1.33 (0.91-1.94) |

| **Women (n=3202)** | | **N** | **Events** | **HR (95%CI)** |
| --- | --- | --- | --- | --- |
| **No metabolic syndrome** | normal weight | 859 | 105 | 1 (Reference) |
|  | overweight | 686 | 94 | 1.07 (0.81-1.42) |
|  | obese | 203 | 29 | 1.02 (0.68-1.55) |
| **Metabolic syndrome** | normal weight | 197 | 41 | 1.41 (0.98-2.03) |
|  | overweight | 689 | 113 | 1.23 (0.94-1.61) |
|  | obese | 568 | 95 | 1.29 (0.97-1.70) |

N, number; HR, hazard ratio; CI, confidence interval. Hazard ratios and 95%CI are for the multivariable model adjusted for age, gender, smoking, total cholesterol, treatment for hyperlipidemia, estimated glomerular filtration rate (GFR), alcohol, physical activity and education.
