# Supplementary material for: Metabolically Healthy Obesity and the Risk of Cardiovascular Disease in the Elderly Population
Source: PLoS One. 2016 Apr 21;11(4):e0154273. doi: 10.1371/journal.pone.0154273 (PMC4839559; doi:10.1371/journal.pone.0154273)
Supplement: S4 Table — Hazard ratios and 95%confidence intervals are presented for the multivariable model adjusted for age, gender, smoking, cholesterol, treatment for hyperlipidemia, estimated glomerular filtration rate (GFR), alcohol, physical activity, education and body mass index. (DOCX) [file pone.0154273.s004.docx]

**S4 Table. Association of metabolic syndrome with cardiovascular disease after adjusting for body mass index.**

|  | **Adjustment** | **N** | **Event** | **HR (95%CI)** |
| --- | --- | --- | --- | --- |
| **No metabolic syndrome** |  | 3038 | 444 | 1 [Reference] |
| **Metabolic syndrome** | BMI, continuous | 2276 | 417 | 1.25 (1.08-1.46) |
|  | BMI, categorical | 2276 | 417 | 1.26 (1.08-1.46) |

Hazard ratios and 95%CI are for the multivariable model adjusted for age, gender, smoking, cholesterol, treatment for hyperlipidemia, estimated glomerular filtration rate (GFR), alcohol, physical activity, education and BMI.
