# Supplementary material for: Metabolically Healthy Obesity and the Risk of Cardiovascular Disease in the Elderly Population
Source: PLoS One. 2016 Apr 21;11(4):e0154273. doi: 10.1371/journal.pone.0154273 (PMC4839559; doi:10.1371/journal.pone.0154273)
Supplement: S5 Table — Hazard ratios and 95%confidence intervals are presented for the multivariable model adjusted for age, gender, smoking, cholesterol, treatment for hyperlipidemia, estimated glomerular filtration rate (GFR), alcohol, physical activity, and education. (DOCX) [file pone.0154273.s005.docx]

**S5 Table. Associations of one, two, three, four, or five components of the metabolic syndrome with cardiovascular disease**

|  | | **N** | **Events** | **HR (95%CI)** |
| --- | --- | --- | --- | --- |
| Number of metabolic syndrome components | None | 433 | 32 | 1 (Reference) |
|  | One | 1153 | 175 | 1.87 (1.28-2.72) |
|  | Two | 1452 | 237 | 1.94 (1.34-2.82) |
|  | Three | 1171 | 200 | 2.08 (1.43-3.02) |
|  | Four | 732 | 134 | 2.35 (1.59-3.46) |
|  | Five | 373 | 83 | 2.99 (1.97-4.51) |

N, number; HR, hazard ratio; CI, confidence interval. Hazard ratios and 95%CI are for the multivariable model adjusted for age, gender, smoking, total cholesterol, treatment for hyperlipidemia, estimated glomerular filtration rate (GFR), alcohol, physical activity and education.
